# Translational changes induced by acute sleep deprivation uncovered by TRAP-Seq

**DOI:** 10.1186/s13041-020-00702-5

**Published:** 2020-12-03

**Authors:** Lisa C. Lyons, Snehajyoti Chatterjee, Yann Vanrobaeys, Marie E. Gaine, Ted Abel

**Affiliations:** 1 Department of Neuroscience and Pharmacology, Iowa Neuroscience Institute, Carver College of Medicine, University of Iowa, Iowa City, IA USA; 2 Program in Neuroscience, Department of Biological Science, Florida State University, Tallahassee, FL USA; 3 Present Address: Department of Pharmaceutical Sciences and Experimental Therapeutics (PSET), College of Pharmacy, University of Iowa, Iowa City, IA USA

**Keywords:** Sleep deprivation, Ribosome, Memory, Hippocampus, TRAP, RNA, Gene expression

## Abstract

Sleep deprivation is a global health problem adversely affecting health as well as causing decrements in learning and performance. Sleep deprivation induces significant changes in gene transcription in many brain regions, with the hippocampus particularly susceptible to acute sleep deprivation. However, less is known about the impacts of sleep deprivation on post-transcriptional gene regulation. To identify the effects of sleep deprivation on the translatome, we took advantage of the RiboTag mouse line to express HA-labeled Rpl22 in CaMKIIα neurons to selectively isolate and sequence mRNA transcripts associated with ribosomes in excitatory neurons. We found 198 differentially expressed genes in the ribosome-associated mRNA subset after sleep deprivation. In comparison with previously published data on gene expression in the hippocampus after sleep deprivation, we found that the subset of genes affected by sleep deprivation was considerably different in the translatome compared with the transcriptome, with only 49 genes regulated similarly. Interestingly, we found 478 genes differentially regulated by sleep deprivation in the transcriptome that were not significantly regulated in the translatome of excitatory neurons. Conversely, there were 149 genes differentially regulated by sleep deprivation in the translatome but not in the whole transcriptome. Pathway analysis revealed differences in the biological functions of genes exclusively regulated in the transcriptome or translatome, with protein deacetylase activity and small GTPase binding regulated in the transcriptome and unfolded protein binding, kinase inhibitor activity, neurotransmitter receptors and circadian rhythms regulated in the translatome. These results indicate that sleep deprivation induces significant changes affecting the pool of actively translated mRNAs.
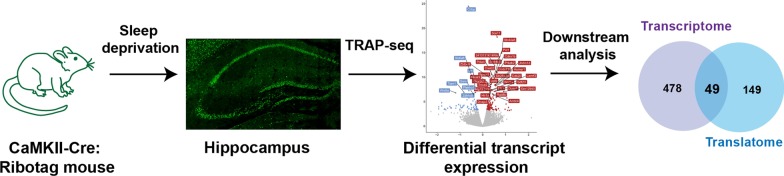

## Introduction

Sleep deprivation is a widespread problem affecting more than one-third of U.S. adults and 70% of teenagers (Center for Disease Control and Prevention, 2017) leading to significant impairments in memory and performance. The effects of sleep deprivation on learning and memory are phylogenetically conserved from invertebrates such as *Aplysia* and *Drosophila* to humans [1–5]. Sleep disorders have been linked to neurodegenerative diseases, including Alzheimer’s disease and Parkinson’s disease, seemingly increasing disease risk and progression (reviewed in [6]; [7–9]). The hippocampus appears particularly susceptible to the effects of acute sleep deprivation with sleep deprivation affecting gene expression, kinase signaling pathways, spatial memory consolidation, synaptic plasticity and neuronal structure [10–17]. The hippocampus is not the only brain region affected by sleep deprivation, as studies of the cortex and forebrain also reported changes in gene expression after sleep deprivation [18–21]. Despite the widespread transcriptional changes induced by sleep deprivation and the severity of the impact on memory, the mechanisms by which sleep deprivation adversely impairs neuronal function have not been fully elucidated.

Acute sleep deprivation causes the transcriptional downregulation of genes involved in mTOR signaling and protein synthesis [10], with subsequent research finding that sleep deprivation decreased mTOR signaling resulting in hypophosphorylated 4EBP2 and significantly decreased protein synthesis [22]. Sleep deprivation appears to impact translation initiation rather than elongation as sleep deprivation decreases the association of the cap binding protein eIF4E with eIF4G [22]. Interestingly, researchers found that the effects of sleep deprivation on protein synthesis and long-term spatial memory were mitigated through overexpression of 4EBP2 in excitatory neurons, suggesting that sleep deprivation primarily affects memory consolidation through suppression of translation in excitatory neurons [22]. We hypothesized that sleep deprivation regulates gene expression at multiple levels from transcription to RNA processing to translation and post-translational modifications. Recent research of synaptic transcripts and proteins in the forebrain lends support to this hypothesis with the finding that sleep deprivation dampens rhythms in the synaptic proteome and eliminates most rhythms in phosphorylation for synaptic proteins [23, 24]. However to date, it has been difficult to identify the subset of genes most affected at the protein level as most studies measuring protein abundance after sleep deprivation in a specific brain region have focused on individual proteins [25, 26],

Translating ribosome affinity purification combined with unbiased RNA sequencing (TRAP-Seq) has become a powerful technique for identification of the translatome, the set of mRNA transcripts bound to ribosomes, in a cell-type specific manner [27–31]. The development of mouse lines with labeled ribosomes has advanced the use of TRAP-Seq for cell-type specific analysis of behavior and disease models [28, 30, 32–35]. In the hippocampus, the CaMKIIα-Cre transgene has been used to specifically express the HA-tagged ribosomal protein Rpl22 at endogenous levels in excitatory neurons [32]. Taking advantage of this technique, we investigated the effects of sleep deprivation downstream of transcription at the level of the ribosome.

After initial verification that the TRAP technique de-enriched for mRNA transcripts in glia and inhibitory neurons with similar results between males and females, we investigated the effects of 5 h sleep deprivation on ribosome-associated RNA. We identified 198 transcripts that were significantly different in abundance in the pool of ribosome-bound mRNA following sleep deprivation, finding 132 upregulated genes enriched for functions in protein processing, unfolded protein response and misfolded protein binding, while the 68 downregulated genes preferentially affected circadian rhythms and voltage-gated potassium channel activity. We determined that 56 of these genes had multiple isoforms, with 54 having one transcript differentially regulated while the other transcript(s) was unchanged after sleep deprivation. Through comparison with previously published hippocampal data on differential gene transcript levels after acute sleep deprivation [10], we found that significant differences exist between total RNA and ribosome-bound transcripts after sleep deprivation with considerably fewer differentially expressed genes detected after sleep deprivation in the TRAP-Seq data. Surprisingly, there were only 49 genes regulated similarly in the transcriptome and the translatome after sleep deprivation. Analysis of the genes that were not differentially expressed at the level of the ribosome, but were in the transcriptome, revealed these genes to be involved in transcription, histone deacetylase activity and small GTPase protein binding. In contrast, the genes that were differentially regulated in the translatome were involved in unfolded protein binding, kinase inhibitor activity and circadian rhythms. Our results highlight the multiple levels through which sleep deprivation impacts gene regulation.

## Materials and methods

### Animals

Female RiboTag mice B6N.129-*Rpl22*^*tm1.1Psam*^/J (Jackson Laboratory Stock 011029) were crossed with male B6.Cg-Tg(CaMKIIα-Cre)T29-1Stl/J (Jackson Laboratory Stock 005359) mice to generate CaMKIIα-Cre:RiboTag progeny with targeted HA-tagged Rpl22 expression in excitatory neurons. Mice were group housed in a 12-h light/12 h-dark cycle room with temperature and humidity controlled (22 °C and 55 ± 5%, respectively) and ad libitum access to food and water. Experiments with male and female mice were conducted independently. All animals were genotyped prior to use in experiments. All experiments at the University of Iowa were approved by the University of Iowa Institutional Care and Use Committee and conducted in accordance to National Institute of Health guidelines.

### Immunohistochemistry

Immunohistochemistry using an antibody to the HA tag was used to confirm the pattern of transgene expression in the CaMKIIα-Cre:RiboTag mice. Following sedation with isoflurane, animals were perfused with PBS and 4% paraformaldehyde in PBS. Whole brains were fixed in 4% paraformaldehyde in PBS at 4 °C for 2 h and then placed in 30% sucrose in PBS for 48–72 h. Cryosectioned slices (30 microns) were stored in well plates in a PBS cryroprotectant solution (150 g sucrose, 150 ml ethylene glycol in total volume of 500 ml). For antibody staining, slices were rinsed three times in PBS, permeabilized with Triton-X, blocked for 1 h with normal goat serum (Invitrogen) at room temperature on a rotating shaker, then incubated overnight at 4 °C with Anti-HA.11 Epitope Tag antibody (Biolegend). Sections were washed three times in PBS, incubated at room temperature for 2 h with the secondary antibody Alexa Fluor 488 goat anti-mouse (Invitrogen), washed three times in PBS and mounted on slides with ProLong Diamond Antifade Mountant with DAPI (Thermofisher). Imaging was done on a Leica SPE Confocal Microscope (Leica).

### Sleep deprivation

Sleep deprivation was performed as previously described using gentle handling [10, 17, 22]. Briefly, 1 week prior to experiments, animals were single housed with corncob bedding with water bottles and ad libitum access to food. Animals were gently handled through tapping of the cages and cage shakes for 3 min for 3 days prior to the scheduled experiment [13, 36]. All sleep deprivation was performed starting at the beginning of the light–dark cycle to avoid circadian confounds. Sleep deprivation was performed for 5 h using gentle handling through cage tapping and then cage shakes as necessary. To ensure that only the minimal amount of disturbance was used to achieve sleep deprivation, animals were monitored continuously. In all experiments, control (non-sleep deprived) animals were sacrificed at the same time as experimental animals. Following sleep deprivation, hippocampi were dissected from experimental and control animals and flash frozen using dry ice.

### TRAP RNA extraction

Ribosome-associated mRNA was immunoprecipitated as previously described [28, 37]. Hippocampi from two mice were pooled and homogenized in 1.25 ml homogenization buffer (50 mΜ Tris–HCl pH 7.4, 100 mΜ KCl, 12 mΜ MgCl_2_, 1% NP40, cycloheximide 100 μg/ml, heparin 1 mg/ml, Halt Protease Inhibitor Thermofisher 10 μl/ml, Promega RNAsin 5 μl/ml, 1 mΜ DTT) on ice. Samples were centrifuged (10 min, 10,000*g*, 4 °C) and then an 80 μl sample from the supernatant was removed for total RNA comparison. The supernatant was incubated with 5 μl Anti-HA.11 Epitope Tag antibody (Biolegend) for 4 h at 4 °C and then with Pierce Protein A/G magnetic beads (Thermofisher) for 17 h incubation at 4 °C. Supernatant was removed from the beads using a magnetic rack on ice. Beads were washed three times (10 min each) with high salt buffer (50 mΜ Tris pH 7.4, 300 mΜ KCl, 12 mΜ MgCl_2_, 1% NP40, cycloheximide 100 μg/ml, 1 mΜ DTT), RNA eluted from the beads using 350 μl of RLT buffer from the RNAeasy Micro kit (Qiagen) and subsequently processed using manufacturer’s directions for RNA extraction. The RNAeasy Micro kit (Qiagen) was also used to elute total RNA from the 80 μl homogenate. DNAse reactions were performed on columns. RNA concentration and quality were initially assessed using a Nanodrop (Thermo Fisher Scientific).

### Library preparation

RNA quality was assessed using a BioAnalyzer (Agilent). RNA library preparation from TRAP samples of sleep deprived (n = 6 samples) and non-sleep deprived (n = 5 samples) mice were prepared at the Iowa Institute of Human Genetics (IIHG), Genomics Division, using the Illumina TruSeq Stranded Total RNA with Ribo-Zero gold sample preparation kit (Illumina, Inc., San Diego, CA). Library concentrations were measured with KAPA Illumina Library Quantification Kit (KAPA Biosystems, Wilmington, MA). Pooled libraries were sequenced on Illumina NovaSeq 6000 sequencers with 150-bp Paired-End chemistry (Illumina) at the IIHG core, resulting in an average of 86,763,851 reads per sample in the non-sleep deprived mice and 90,710,358 reads per sample after sleep deprivation. The dataset supporting the conclusions of this article is available in the NCBI’s Gene Expression Omnibus repository, GEO Series accession GSE156925.

### TRAP-Seq analysis

Sequencing data was processed with the reads trimmed, aligned and quantified according to the MUGQIC RNA-seq pipeline (bitbucket.org/mugqic/genpipes/src/master/pipelines/rnaseq). Briefly, raw reads quality trimming and removing of Illumina adapters was performed using Trimmomatic v0.36 with parameters ILLUMINACLIP:adapters-truseq.fa:2:30:15:8:true TRAILING:30 MINLEN:32 [38]. The remaining reads were then aligned to the Mus musculus reference genome (mm10) with STAR v2.7.5 [39] and a raw count matrix for all the samples was generated using HTSeq-counts in mode—intersection-nonempty with the default parameter—nonunique none. Therefore the reads that overlap more than one feature are counted as ambiguous and not counted for any features [40]. All further analyses were performed using R version 3.6.0 [41]. We eliminated any genes that were not detected to increase rigor of analysis. For gene level count data, the R package EDASeq was used to normalize for sequencing depth through upper quartile normalization. Differential expression analysis was conducted using edgeR [42] and adjusted for multiple comparisons. Analysis code available through Github at https://github.com/YannVRB/Sleep-deprivation-and-protein-translation-TRAP-seq.git.

Enrichment analysis of DEG-associated pathways and molecular functions from the microarray or the TRAP data was performed with a combination of Kyoto Encyclopedia of Genes and Genomes (KEGG) and the Gene Ontology (GO–molecular function-EBI-Uniport-GOA-) databases. The analyses were done with the Cytoscape (version.3.8.0, https://cytoscape.org/) [43] plug-in ClueGO (version 2.5.6) [44]. Only the pathways with a p-value < 0.05 and gene counts ≥ 3 were considered as significant and displayed. To connect the terms in the network, ClueGO utilizes kappa statistics in which here was set as ≥ 0.4. PANTHER Molecular function was used for enrichment analysis of the 49 common genes (Fig. 3). Data was visualized using NetworkAnalyst (www.networkanalyst.ca). Chord diagram was generated and visualized using NetworkAnalyst.

### Quantitative real-time PCR

cDNA was prepared from 100 to 400 ng of RNA using Superscript IV First-Strand Synthesis (Ambion) according to manufacturer’s instructions using random hexamers. Reverse transcriptase free reactions and RNA absent reactions were performed as controls. qPCR was performed using SybrGreen Master Mix (Thermo Fisher Scientific) with reactions run using the Quant Studio 7 Real-Time PCR System (Thermo Fisher Scientific) using specific primers (Additional file 2: Table S5).

### Statistical analyses

All statistical analyses were performed using Graphpad Prism. IP enrichment was analyzed using a mixed effect model with Sidak post-hoc analysis. Unpaired Student’s t-test was applied for qPCR validations of gene expression. Results were expressed as means ± SEM. Values of p < 0.05 were considered as statistically significant.

## Results

### Isolation of ribosome-associated RNA transcripts from hippocampal CaMKIIα expressing neurons

**Fig. 1:**
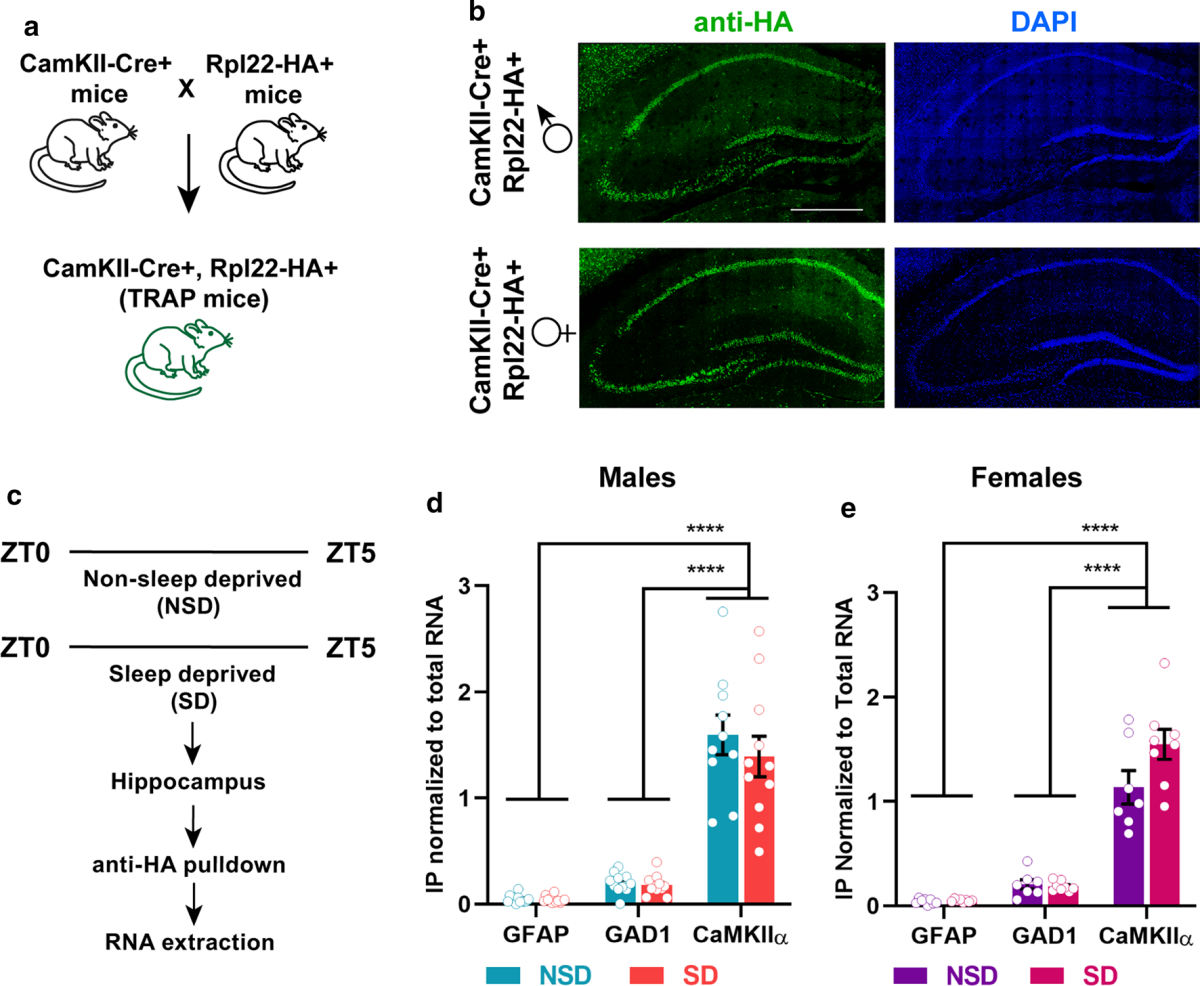
TRAP approach efficiently enriches for ribosome-associated mRNA transcripts from excitatory neurons in the mouse hippocampus. **a** CaMKIIα-Cre mice were crossed with Cre responsive Rpl22-HA mice. Male and female progeny (CaMKIIα-Cre:RiboTag) were used for all subsequent experiments. **b** Immunofluorescence images using anti-HA antibody (green) and DAPI (blue) from CaMKIIα-Cre:RiboTag mice shows expression of Rpl-22-HA expression in mouse hippocampus from male and female mice aged 2–3 months. All images have the same magnification with the scale bar shown indicating 500 microns. **c** CaMKIIα-Cre:RiboTag mice were either sleep deprived for 5 h (from ZT0 to ZT5) or non-sleep deprived, and hippocampus were dissected and processed for ribosome affinity purification of transcripts using anti-HA antibody. **d**, **e** qPCR analysis of immuno-pulldown efficiency revealed enrichment of *CaMKIIα* (excitatory neuronal marker gene) compared to GAD1 (interneuron marker gene) and *GFAP* (glial marker gene) in CamKIIα-Cre:RiboTag males (**d**) and females (**e**). Males: Mixed-effect analysis revealed no significant interaction between “Condition (SD or NSD)” and “Genes (IP enrichment)” F (2, 39) = 0.6336, p = 0.5361. Significant main effect of “Genes” F (1.024, 19.97) = 131.9, p < 0.0001 was observed, while no significant main effect of “condition” F (1, 22) = 0.6307, p = 0. 0.4356 was observed. Sidak multiple comparisons revealed significant difference between: *GFAP* vs. *CaMKIIα*: p < 0.0001, *GAD1* vs. *CaMKIIα*, p < 0.0001. Females: Mixed-effect analysis revealed significant interaction between “Condition (SD or NSD)” and “Genes” F (2, 26) = 4.338, p = 0.0237. Significant main effect of “Genes”: F (1.055, 13.72) = 149.9, p < 0.0001, while no significant main effect of “Condition”: F (2, 39) = 0.6336, p = 0.5361 was observed. Sidak multiple comparisons revealed significant difference between: *GFAP* vs. *CaMKIIα*, p < 0.0001, *GAD1* vs. *CaMKIIα*, p < 0.0001

To isolate ribosome-associated mRNA transcripts associated in excitatory neurons, we used the RiboTag mouse with ribosomal protein Rpl22 labeled with a hemagglutinin epitope ([28]; Jackson Laboratory Stock 011029) crossed with CaMKIIα mice (Jackson Laboratory Stock 005359) to generate mice with expression of the HA-tagged Rpl22 in neurons expressing *CaMKIIα* (Fig. 1a), a technique previously used to identify translating mRNAs during hippocampal synaptic plasticity [32]. The specificity of the expression of the Rpl22-HA tag was verified using immunohistochemistry with an anti-HA antibody in both male and female mice (2–3 months of age, Fig. 1b). As expected, the HA tag appeared almost exclusively in excitatory neurons in both male and female mice. Following immunoprecipitation of whole hippocampal homogenates with an anti-HA antibody and RNA extraction, we compared the TRAP RNA samples with total RNA input samples to determine immunoprecipitation efficiency. We performed qPCR using *GFAP* as a marker for glial cells and *GAD1* as a marker for inhibitory neurons, finding that the TRAP samples were significantly de-enriched for *GFAP* and *GAD1* compared to total input RNA samples, whereas *CaMKIIα* remained high (Fig. 1d, e). We confirmed that sleep deprivation through gentle handling for 5 h (Fig. 1c) did not affect the de-enrichment of glial cells and inhibitory neurons in the TRAP samples with no differences seen for marker genes between samples prepared from sleep deprived or non-sleep deprived animals (Fig. 1d, e).

### Sleep deprivation induces changes in the subsets of RNA transcripts associated with ribosomes

**Fig. 2:**
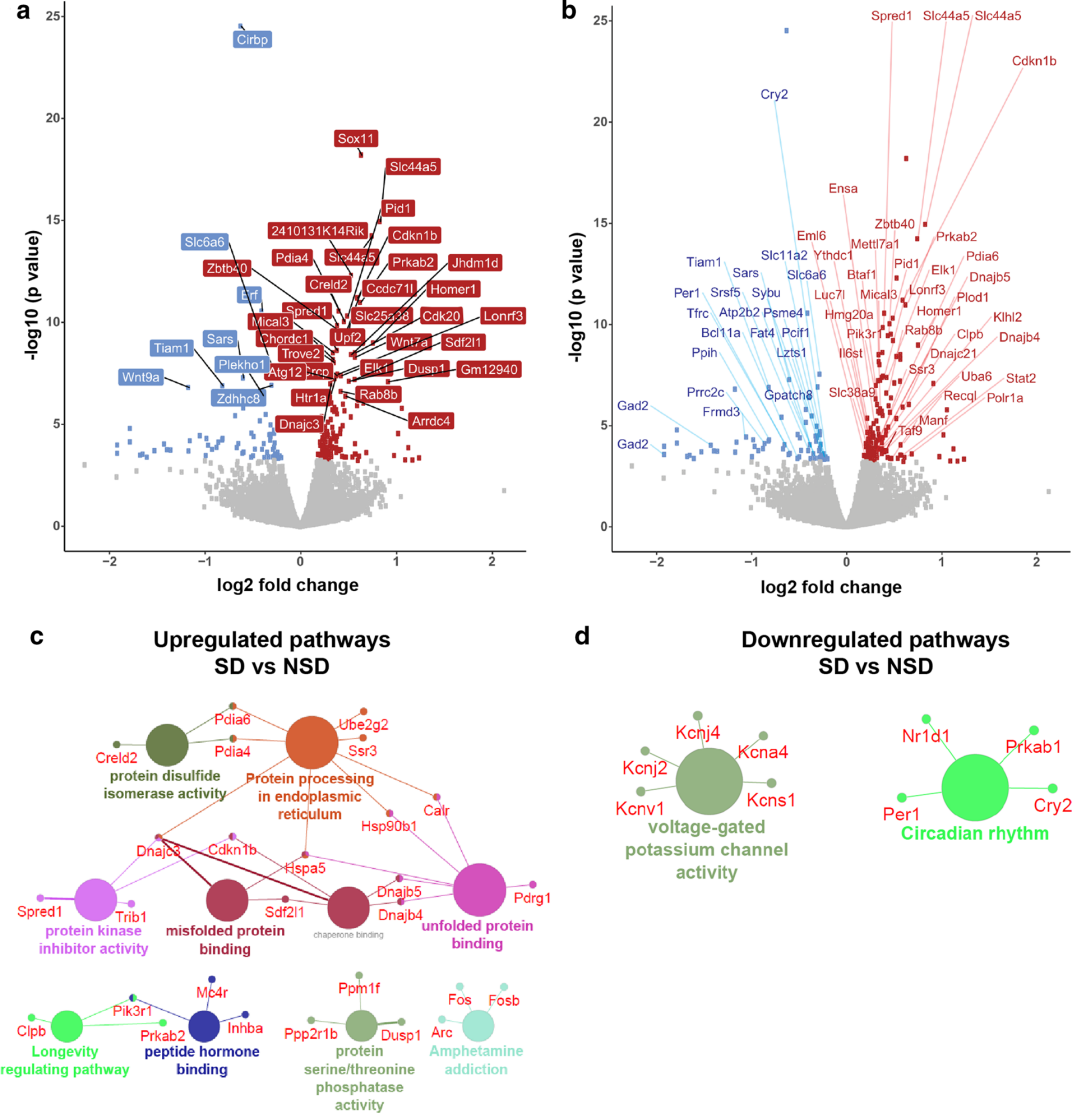
TRAP-Seq reveals differential transcript abundance in the translatome following sleep deprivation. **a** TRAP RNA extraction followed by RNA-seq from the whole hippocampus of sleep deprived and non-sleep deprived male mice (2 pooled per sample) revealed differential expression of ribosome-associated mRNA transcripts. Volcano plot with a threshold of FDR < 0.05 of the transcripts significantly differentially expressed (up-regulated in red, down-regulated in blue, non-significant in grey). The 20 most significantly differentially expressed genes (DEGs) are labeled. **b** Volcano plot showing differential expression of transcript variants. **c**, **d** Pathway analysis (KEGG and GO:molecular function terms) of all the upregulated genes were involved in the protein processing in ER, unfolded protein binding, misfolded protein binding, protein kinase inhibitor activity among the upregulated pathways (**c**), while voltage gated potassium channel activity and circadian rhythms were among the downregulated pathways (**d**)

To determine whether sleep deprivation induced differential expression of mRNA transcripts in the translatome, we performed acute sleep deprivation from ZT 0 to ZT 5 on CaMKIIα-Cre:RiboTag male mice (2–3 months old). Hippocampi from sleep deprived and non-sleep deprived mice were dissected at the same time to avoid differences in gene expression due to circadian regulation (Fig. 1c). Following immunoprecipitation and extraction of TRAP RNA (2 animals pooled per sample), we used an unbiased RNA sequencing approach and the MUGQIC RNA-seq pipeline [45] to identify and analyze differential gene expression. Using a stringent criterion for significance (False Discovery Rate (FDR) < 0.05) to avoid false positives, we found differentially expressed transcripts representing 200 genes, with 132 significantly upregulated and 68 significantly downregulated (Fig. 2a, Additional file 2: Table S1). Of note, considerably fewer genes were significantly impacted by sleep deprivation at the level of translation than would be predicted based on previous research reporting the effects of sleep deprivation on gene transcription [10, 19, 46].

For many genes, including those associated with learning and memory or sleep, alternative splicing generates multiple mRNA transcripts [47–50]. However, it can be difficult to determine whether the products of differential splicing seen at the transcript level are translated. TRAP-Seq facilitates the identification of alternative splicing transcripts that may have functional significance as these transcripts are bound to ribosomes. We analyzed the differentially expressed genes following sleep deprivation using the TRAP data set to identify alternative transcripts associated with the ribosomes. We found that 56 of the differentially expressed genes have multiple isoforms (Fig. 2b, Additional file 2: Table S2). Interestingly, the vast majority of these genes (95%) had one transcript that was differentially expressed after sleep deprivation while the other isoform(s) were not regulated by sleep deprivation. We did not find any genes for which different transcripts were regulated in opposite directions after sleep deprivation. These results are consistent with the hypothesis that sleep deprivation specifically regulates a subset of genes downstream of transcription resulting in changes in mRNA translation.

We separately analyzed the upregulated and downregulated transcripts after sleep deprivation using KEGG and the GO–molecular function (GO:MF) terms to determine potential molecular functions and pathways (Fig. 2c, d). Genes for the ribosome-associated mRNA transcripts upregulated by sleep deprivation include pathways involved in protein processing, misfolded protein binding and the unfolded protein response. Interestingly, we found two major clusters of downregulated genes, those associated with voltage-gated potassium channel activity and those associated with circadian regulation.

### Impacts of sleep deprivation on gene regulation differ between the transcriptome and the translatome

**Fig. 3:**
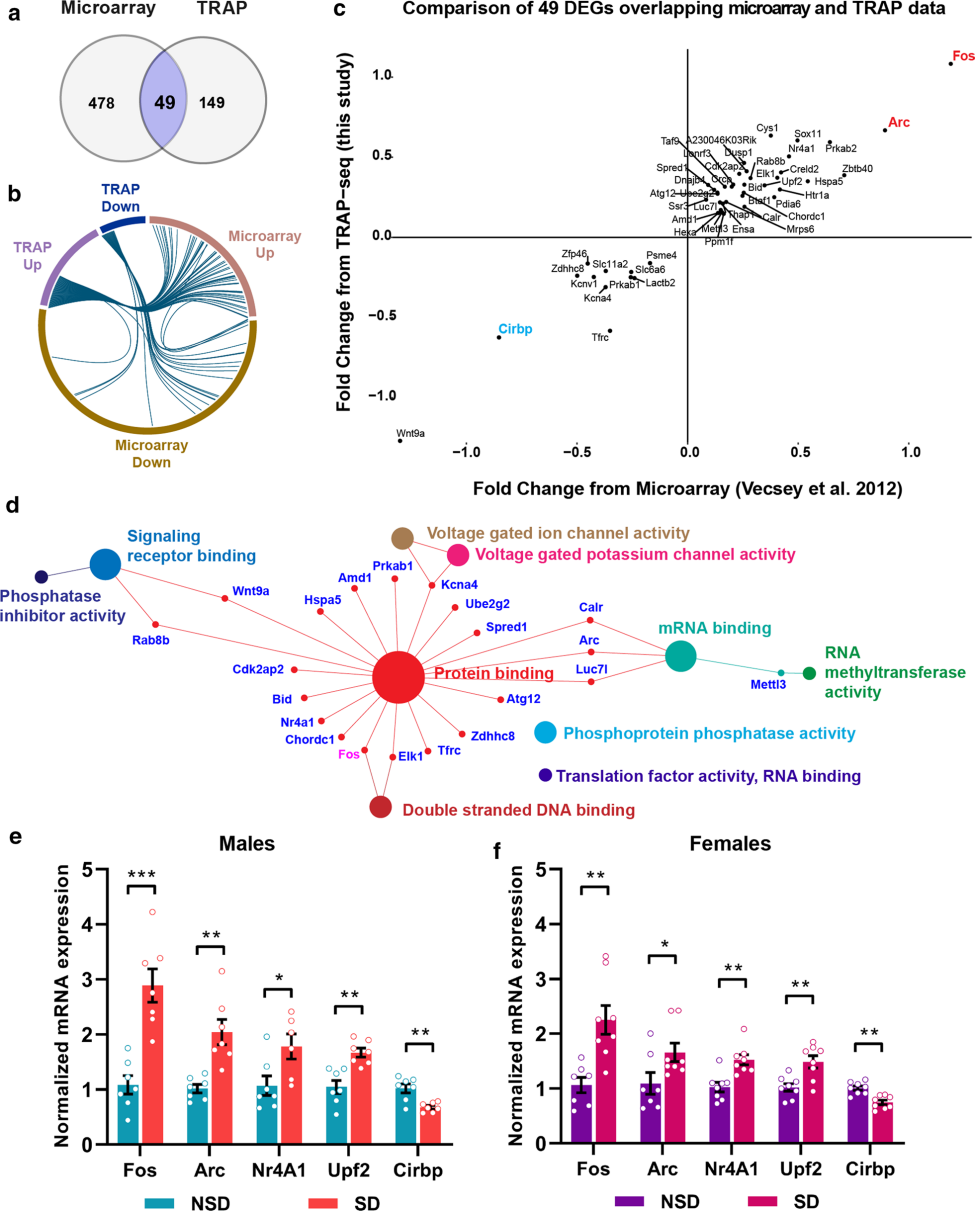
A limited number of genes are impacted similarly by sleep deprivation in the transcriptome and translatome. **a** Venn diagram representing the number of genes differentially expressed between total RNA (microarray) and ribosome-associated transcripts (TRAP) after SD identified 49 common genes, 478 genes only in microarray and 149 genes only in TRAP study. **b** Chord diagram showing comparison of gene expression between TRAP and microarray data from total RNA [10]. **c** Quadrant scatter plots showing the fold change (log2) of the 49 common genes differentially expressed between TRAP and microarray. **d** Pathway analysis of the 49 common genes using PANTHER Molecular function terms showing significantly enriched pathways. All the pathways displayed have a p-value < 0.05. **e**, **f** qPCR validation from independent cohort of animals shows increased expression of *Fos*, *Arc*, *Nr4A1* and *Upf2*, while downregulation of *Cirbp* in males (**e**) and females (**f**) upon sleep deprivation (SD) compared to non-sleep deprived mice (NSD). Unpaired t-test comparing SD vs NSD, males: *Fos*: p = 0.0002, *Arc*: p = 0.0011, *Nr4A1*: p = 0.0285, *Upf2*: p = 0.0010 and *Cirbp*: p = 0.0020, females: *Fos*: p = 0.0023, *Arc*: p = 0.0480, *Nr4A1*: p = 0.0016, *Upf2*: p = 0.0033 and *Cirbp*: p = 0.0005

To determine whether sleep deprivation affects the subset of mRNAs associated with the ribosome independent of the effects of sleep deprivation on gene transcription, we performed a comparative analysis of differential gene expression after acute sleep deprivation from our TRAP-Seq experiments with a previously published data set from our lab of differential gene expression in the whole hippocampus after acute sleep deprivation [10]. This microarray data set (GEO accession GSE33302) was used for comparison as the method of handling prior to the experiment and during sleep deprivation was performed similarly. Although the timing of sleep deprivation was different for the microarray experiments with sleep deprivation performed for five hours starting between ZT 4 and ZT 6, the microarray data set was verified, in part by Vescey and colleagues through qPCR following experiments in which sleep deprivation was performed from ZT 0 to ZT 5 [10]. We analyzed the microarray data set from GEO using the available GEO2R function as has been done in other recently published studies [51, 52] to find differentially expressed genes and genes not affected by sleep deprivation. Surprisingly, we found only 49 genes that were differentially expressed after sleep deprivation in common between the two data sets (Fig. 3a) suggesting that sleep deprivation independently impacts the downstream steps of RNA processing and translation. It should be noted that the microarray transcriptome data set was based upon gene expression from the whole hippocampus including glial cells and inhibitory neurons, therefore, it may not be completely unexpected that the microarray data set contains a greater number of differentially expressed genes. As illustrated through a Chord Diagram, the range of up and downregulation appeared narrower in the TRAP data set than in the whole transcriptome (Fig. 3b). Comparison of the common differentially regulated genes between both data sets revealed that the direction of change was maintained at the total RNA and the TRAP levels (Fig. 3c). Pathway analysis of the common gene set using PANTHER:MF suggests that these genes are primarily protein binding genes and activity dependent genes such as *Arc* and *Fos* (Fig. 3d). We performed independent sleep deprivation experiments in young adult female CamKIIα-Cre:Ribotag mice (2.5–3.5 months) and in a second cohort of slightly older adult male CamKIIα-Cre:Ribotag mice (4.5–7 months) and prepared TRAP RNA samples to confirm the findings from the TRAP-Seq experiments. We performed qPCR for five genes (*Fos*, *Arc*, *Nr4A1*, *Upf2* and *Cirbp*) that were differentially regulated in both the TRAP and the microarray data sets confirming the differential regulation in the TRAP data set (Fig. 3e, f). Although the magnitude of difference between sleep deprived and non-sleep deprived samples appears smaller in females, all genes were significantly different with the same directional change between male and female samples. Furthermore, we confirmed the upregulation of *Arc* following sleep deprivation in the hippocampal transcriptome using the total input RNA from the samples prepared from sleep deprived and non-sleep deprived young adult male mice that were used for the TRAP-Seq experiments (Additional file 1: Figure S1).

### Impacts of sleep deprivation unique to the transcriptome

**Fig. 4:**
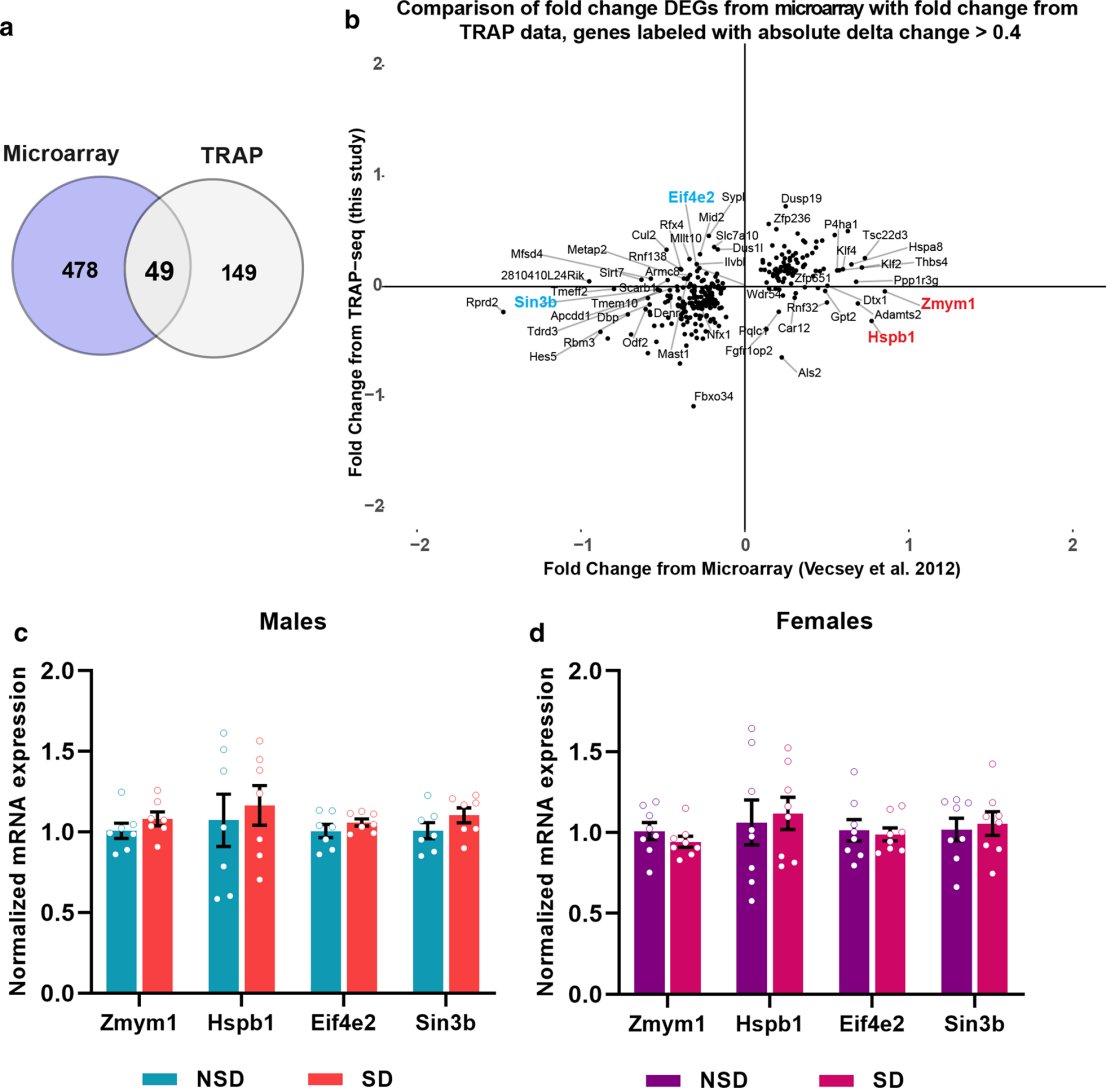
Many genes are regulated by sleep deprivation in the transcriptome but not in the translatome. **a** Venn diagram showing overlap of differentially expressed genes between TRAP and microarray after SD. **b** Quadrant scatter plots showing the fold change (log2) of DEGs exclusive from microarray with their respective fold change from TRAP (genes that are only responsive at the total RNA level but not at the level of ribosome-associated transcripts). The genes in the outermost layer are labeled with absolute delta fold change (FC_microarray_ − FC_TRAP_) value above 0.4. **c**, **d** q-PCR validation of some of the genes associated with transcriptional repression after SD in males (**c**) and females (**d**). Unpaired t-test comparing SD vs NSD, males: *Zmym1*: p = ns, *Hspb*1: p = ns, *Eif4e2*: p = ns and *Sin3b*: p = ns, females: *Zmym1*: p = ns, *Hspb1*: p = ns, *Eif4e2*: p = ns and *Sin3b*: p = ns

The finding that only a comparatively small number of genes were similarly regulated after sleep deprivation in the transcriptome and translatome raises additional questions. What are the genes regulated dissimilarly at the level of transcription or translation by sleep deprivation and do these discrete gene sets implicate distinct biological functions? In total, we found 478 genes differentially regulated in the hippocampal transcriptome [10] that did not appear in the list of differentially expressed transcripts in the TRAP-Seq data (Fig. 4a). However, as the TRAP-Seq protocol enriches for excitatory neurons, we would expect that genes exclusively expressed in glia or inhibitory neurons would not be present in the TRAP-Seq data. We found 280 genes that were differentially expressed in the transcriptome that were present in the TRAP-Seq data set at similar levels between experimental and control groups. We performed a more in-depth analysis of genes regulated only at the level of transcription focusing on genes with a more than 0.4 log_2_ absolute fold change in expression between the microarray and TRAP data set (Fig. 4b, Additional file 2: Table S3). We found numerous genes that were significantly upregulated or downregulated in the transcriptome after sleep deprivation, yet similar in abundance between sleep deprivation and control samples when the TRAP data was analyzed. Potentially, low mRNA abundance in the TRAP data set for some genes resulted in genes appearing non-significant in the TRAP-Seq data set or highly variable. Consequently, we used qPCR with gene specific primers to determine whether some of the most significant differentially expressed genes (with high fold changes) from the microarray were differentially expressed in TRAP RNA samples (Fig. 4c, d). Using samples from independent experiments for males and females, we found that *Zmym1*, a zinc finger protein, which shows greater than 0.85 log_2_ fold upregulation in the whole transcriptome, showed no difference when ribosome-associated mRNA transcripts were measured. Similarly, no change was found in the TRAP samples for the molecular chaperone *Hspb1* that shows 0.77 log_2_ fold increase in the transcriptome. We confirmed the regulation by sleep deprivation in the transcriptome for *Hspb1* using the total RNA samples from the sleep deprived and non-sleep deprived young males for which TRAP-Seq was performed finding that *Hspb1* was significantly increased with sleep deprivation in the hippocampal transcriptome (Additional file 1: Figure S1). We also specifically tested whether the downregulation of the transcriptional repressor *Sin3b* with − 0.53 log_2_ fold change in the transcriptome was unchanged in the TRAP samples finding that *Sin3b* was equally expressed in both sleep deprivation and control conditions. Potentially, for some genes, such as those for which mRNA transport and localization occurs, only a fraction of the total mRNA transcripts may be actively translated. These results strongly suggest that sleep deprivation affects gene expression post-transcriptionally by influencing the association of specific mRNA transcripts with ribosomes.

### Gene regulation in the translatome after sleep deprivation

**Fig. 5:**
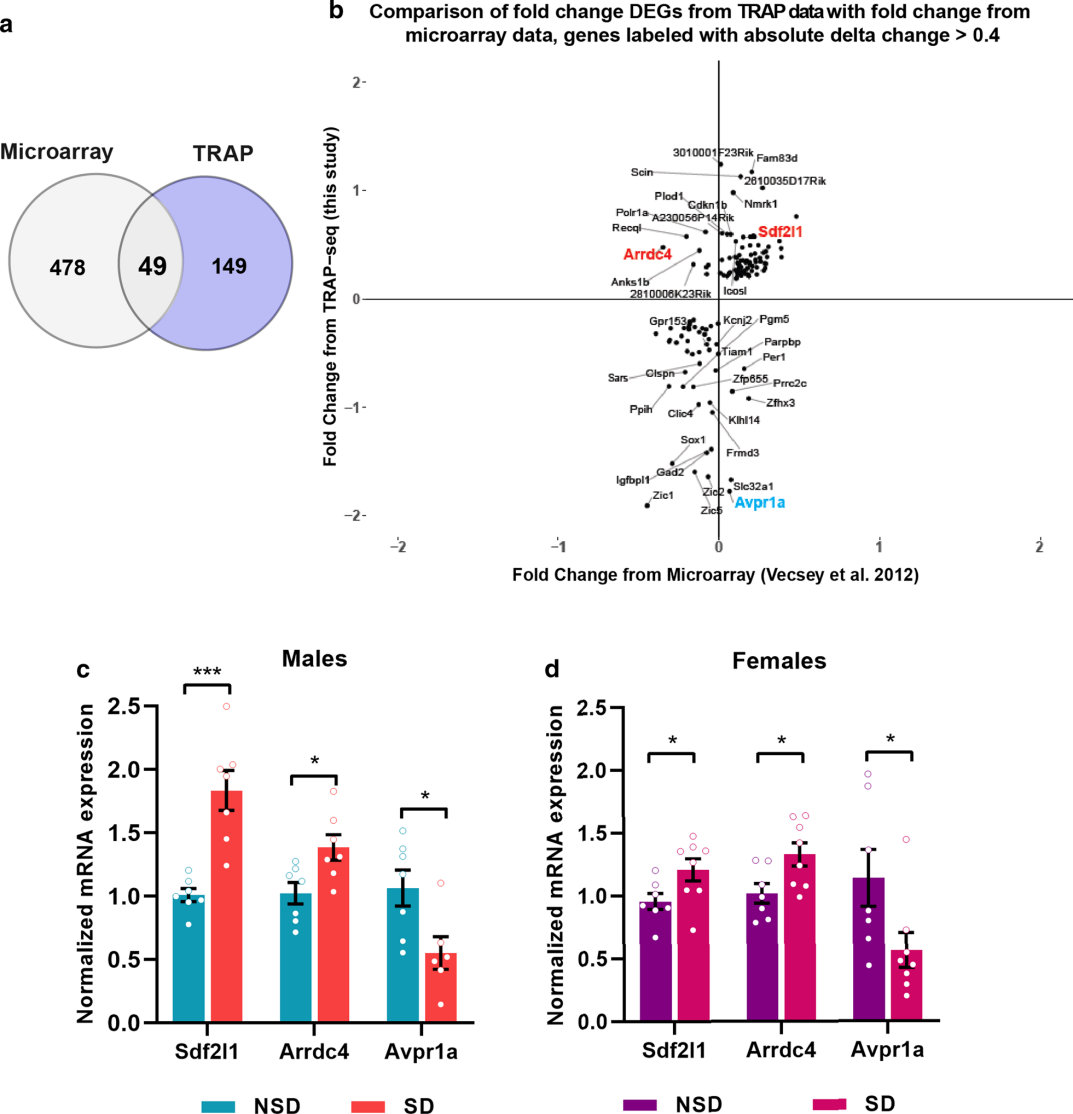
Genes exclusively responsive to sleep deprivation in the translatome. **a** Venn diagram showing overlap of differentially expressed genes between TRAP and microarray after SD. **b** Quadrant scatter plots showing the fold change (log2) of DEGs exclusive from TRAP with their respective fold change from microarray (genes that are only responsive at the level of ribosome-associated transcripts but not total RNA). The genes in the outermost layer are labeled if their absolute delta fold change (FC_TRAP_ − FC_microarray_) value is above 0.4. **c**, **d** q-PCR validation of some of the genes associated with protein folding and Ubiquitinylation after SD in males (**c**) and females (**d**). Unpaired t-test comparing SD vs NSD, males: *Sdf2l1*: p = 0.0003, *Arrdc4*: p = 0.0171, *Avpr1a*: p = 0.0229. Females: *Sdf2l1*: p = 0.0423, *Arrdc4*: p = 0.0258, *Avpr1a*: p = 0.0445

We also wanted to identify genes that were not differentially regulated by sleep deprivation in the transcriptome, but were altered in the TRAP samples as this would indicate a distinct level of regulation targeted by sleep deprivation. We found 149 genes that were significantly regulated in the TRAP data set that were not significantly different in the transcriptome (Fig. 5a, Additional file 2: Table S4) strongly indicating post-transcriptional regulation with sleep deprivation. We verified the changes in the TRAP-Seq data set for genes of interest in this group using qPCR (Fig. 5c, d). We found that *Sdf2l1*, a chaperone protein associated with protein misfolding, was significantly upregulated in the TRAP samples in males and females as was the ubiquitin protein ligase adapter *Arrdc4*. We verified the lack of differential expression of *Arddc4* after sleep deprivation at the level of the transcriptome through qPCR experiments from the total RNA samples of the young males for which TRAP-Seq was performed (Additional file 1: Figure S1). Of interest, the arginine vasopressin receptor *Avpr1a* that has been implicated in synchronization of circadian circuits [53, 54] was significantly downregulated in the TRAP samples after sleep deprivation. These results provide insight into some of the potential impacts associated with sleep deprivation including increased protein misfolding, protein degradation and misalignment of circadian rhythms.

**Fig. 6:**
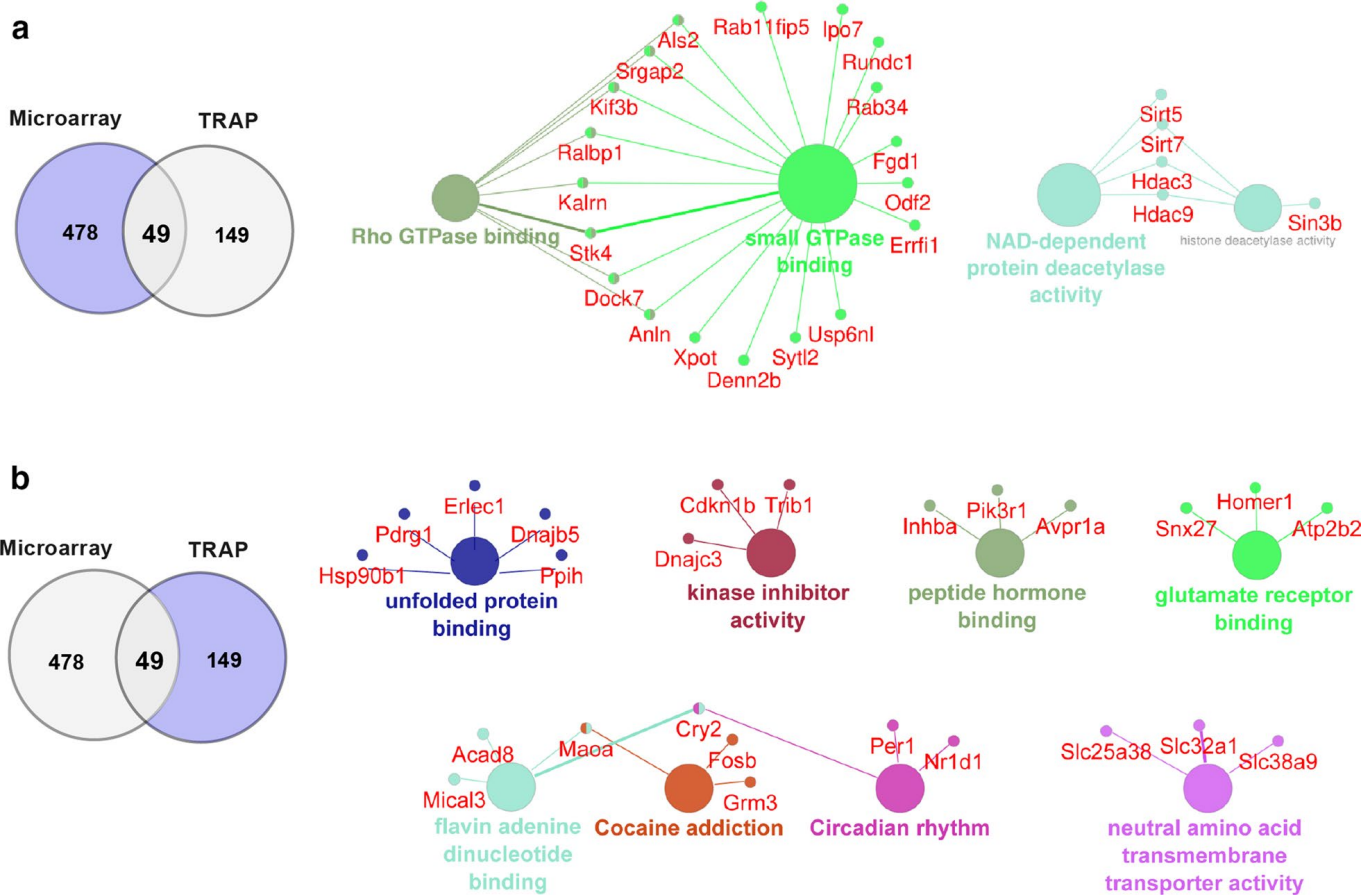
Differences in profile signatures between genes impacted by sleep deprivation in the transcriptome and translatome. All the pathways displayed have a p-value < 0.05. **a** Pathway analysis (KEGG and GO: molecular terms) of the DEGs regulated by sleep deprivation only in the transcriptome identified small GTPase binding, NAD-dependent protein deacetylase activity and histone deacetylase activity (Microarray data set from [10]). **b** Pathway analysis (KEGG and GO: molecular terms) of the DEGs exclusively regulated by sleep deprivation in the ribosome-associated mRNA transcripts include unfolded protein binding, circadian activity, neural amino acid transmembrane transporter activity, kinase inhibitor activity and others

To provide a more wide-ranging outlook of the genes regulated by sleep deprivation separately in either the transcriptome or the translatome, we performed pathway analysis of these differentially expressed genes using KEGG and GO:MF (Fig. 6). We found striking differences in the types of pathways for which genes were regulated at the transcriptome and translatome levels. Analysis of the differentially expressed genes from the transcriptome revealed genes involved in transcription, histone deacetylase activity and Rho GTPase binding. In contrast, when the genes were analyzed that exhibited differential expression only in the translatome, these genes were associated with the unfolded protein response, circadian rhythms, and cellular signaling responses including kinase inhibitor activity. These contrasting profiles suggest that the effect of sleep deprivation on the transcriptome and the translatome have different biological functions.

## Discussion

### Multiple levels of gene regulation by acute sleep deprivation

Gene regulation occurs at multiple levels within the cell from chromosome positioning and chromatin accessibility, to transcriptional regulation through transcription factors and enhancers, to post-transcriptional processes and translation [55–57]. Epigenetic and transcriptional regulation of gene expression has received considerable attention in the fields of sleep research [24, 58]; (reviewed in [59]). However, numerous cellular processes regulate gene expression and protein activity downstream of transcription including RNA processing steps such as RNA splicing, nuclear export, RNA localization, RNA availability, translational regulation through initiation and elongation steps, and post-translational modifications. In contrast to transcription, comparatively few studies have investigated the effects of sleep deprivation on these downstream steps. Recent research investigating sleep deprivation has revealed layers of post-transcriptional gene regulation including region specific changes in gene expression [60, 61], mRNA localization [62] and changes in translation [10, 22, 24]. Acute sleep deprivation decreases hippocampal mTOR signaling resulting in lower levels of phosphorylated 4EBP2 and decreased protein synthesis [22]. In its active non-phosphorylated form, 4EBP2 binds to the initiation factor eIF4E through structural mimicry of eiF4G thereby preventing the binding of eIF4E to eIF4G and subsequently hindering cap-dependent translation [63–66]. When phosphorylated by mTOR, the 4EBP2-eIF4E complex dissociates allowing eIF4E to bind eIF4g and the 5′ m7G mRNA cap (reviewed in [67]). Moreover, the decrease in protein synthesis by sleep deprivation appears specific to cap-dependent translation as a second path to translation from mTOR through S6Kinase appears unchanged [22]. Increasing protein synthesis through 4EBP2 overexpression in hippocampal excitatory neurons mitigated the impacts of sleep deprivation on protein synthesis and hippocampus-dependent spatial memory [22]. As sleep loss has been linked to multiple diseases including neurodegenerative diseases and cognitive decrements, it is imperative to understand the full effects of sleep deprivation on gene regulation. Consequently, we took advantage of TRAP-Seq techniques to identify the impact of sleep deprivation on the translatome in the hippocampus.

### Effects of sleep deprivation on translation in excitatory neurons

To answer the question of whether sleep deprivation targets a specific subset of mRNA transcripts, we used CaMKIIα-Cre:RiboTag mice to preferentially enrich for ribosome-associated mRNA transcripts in excitatory neurons combined with an unbiased sequencing approach. TRAP-Seq has been successfully used to understand differential gene translation in hippocampal LTP, with researchers finding significantly more genes differentially expressed in TRAP-Seq in excitatory neurons compared to the whole hippocampus [32]. In contrast, we found that considerably fewer genes were differentially expressed after sleep deprivation when ribosomal bound mRNAs were analyzed compared to the whole hippocampal transcriptome, with 132 genes upregulated and 68 genes downregulated in the TRAP-Seq data set. In addition to the possibility of distinct mechanisms for gene regulation by sleep deprivation in the transcriptome and translatome, variation in the differentially expressed genes between the transcriptome and translatome could arise from several sources. If sleep deprivation triggered similar effects on gene regulation at the transcriptome and translatome levels, one would predict that the differentially regulated genes in our TRAP-Seq studies would be a subset of the differentially expressed genes found in the hippocampal transcriptome used for comparison as total hippocampal tissue also contains inhibitory neurons as well as glial cells. Acute sleep deprivation has been previously shown to affect gene expression in astrocytes and oligodendrocytes in the cortex affecting glial function, apoptosis and differentiation [68–71]. Additionally, long non-coding RNA molecules would also be excluded from the TRAP-Seq data set. The regulation of sleep and sleep deprivation has been previously shown to affect the expression of long non-coding RNAs in vertebrate and invertebrate models [72, 73]. Closer analysis of the microarray transcriptome differentially expressed genes from Vecsey and colleagues [10] found that 22 genes were glial or non-neuronal, and 108 were long non-coding RNAs; thus, accounting for at least part of the lower than predicted numbers of differentially expressed genes we found in the TRAP-Seq data.

Another source of potential differences between the published hippocampal microarray transcriptome data set and the TRAP-Seq data set could arise from the time in which sleep deprivation was performed in each of these studies. In our studies as in Tudor et al. in which sleep deprivation was found to decrease protein synthesis, sleep deprivation was performed for 5 h starting at the beginning of the light cycle, while in the trascriptome study by Vecsey and colleagues that initially found changes in mTOR signaling and downregulation of genes involved in translation, sleep deprivation was started between ZT 3 and ZT 6 continuing for 5 h. However, it should be noted that Vecsey and colleagues verified a subset of the genes of interest using qPCR from sleep deprivation experiments that were conducted from ZT 0 to ZT 5 [10]. Differences in the timing of sleep deprivation does have the potential to affect gene expression levels as many genes are transcriptionally regulated by the circadian clock. However, in both the Vecsey study and our study, tissue from non-sleep deprived animals was collected at the same time as the tissue from sleep deprived animals so that circadian differences in baseline gene expression levels are accounted for within each study. Due to the differences in timing of sleep deprivation and the different gene expression detection tools, we only compared the genes identified by differential analysis from each study and not the basal or induced levels of those genes, thus minimizing errors induced by differences in gene expression due to circadian time. Thus, our results suggest that sleep deprivation can have independent impacts on the transcriptome and the translatome.

Alternative mRNA splicing can be found in all cells generating multiple transcript isoforms from single genes. Alternative splicing of transcripts may affect mRNA stability, RNA localization, or protein interactions (reviewed in [74, 75]). Changes in alternative splicing or splicing dysregulation have been observed in neurodegenerative diseases [75–77]. However, it is difficult to determine whether alternatively spliced transcripts are translated. By using TRAP-Seq, we were able to identify alternatively spliced mRNA transcripts associated with ribosomes, suggesting that these transcripts may have functional significance. Among the genes that we identified as differentially expressed, we found 56 that had multiple isoforms with 54 of these genes having one transcript isoform was differentially regulated by sleep deprivation while the remaining isoform(s) were constitutively expressed. For example, we found that *Homer1* displayed overall differential gene regulation with the shorter transcript (*Homer1–204*, aka *Homer1a*) accounting for the significant difference in expression, while the longer *Homer1–203* (aka isoform L) was unchanged. We also found that one transcript of *Per1, (Per 1–202)* was significantly downregulated in the TRAP-Seq data, while a second isoform remained unchanged by sleep deprivation (*Per1–206*). This is interesting as the *Per1–202* translation start site is located more 5′ than observed for the other *Per1* transcripts, even though the *Per1–202* transcript codes for a shorter protein. In addition to potential differences due to cap-dependent translation, one could speculate that mRNA localization of specific isoforms could also influence transcript association with ribosomes, especially for synaptic proteins.

### Regulation of genes by sleep deprivation in both the transcriptome and translatome

We found that 49 genes were commonly regulated between previously published transcriptomic research [10] and our TRAP-Seq data with directional changes maintained from the transcriptome to the translatome including immediate early response genes. Increased expression of *Arc* following sleep deprivation in the cortex has been recently implicated in sleep homeostasis and important in the induction of other genes following sleep deprivation including *c-fos*, *Homer1a*, *Nur77/NR4A1* and *hspa5/BiP* [62]. Consistent with previous studies of the transcriptome after sleep deprivation in the cortex and the hippocampus [10, 26, 61, 78], we found that *Cirbp* was one of the most downregulated genes in the translatome. Based on studies in the cortex, CIRBP has been hypothesized to act as a link to changes in circadian gene expression following sleep deprivation [78]. Core clock genes may function in sleep homeostasis with sleep deprivation induced changes in circadian genes proposed to increase sleep pressure and recovery sleep [78].

In contrast to previous research by Tudor et al. demonstrating that Arc protein abundance was unchanged in the hippocampus after 5 h of acute sleep deprivation [22], we found the increase in *Arc* mRNA following sleep deprivation was maintained in the TRAP mRNA fraction in young males. We confirmed these results with a second set of independent experiments in males and a set of sleep deprivation experiments in females finding that sleep deprivation significantly increased the abundance of ribosome bound *Arc* mRNA in all groups. On the surface, these results may seem inconsistent with the results of Tudor and colleagues [22]. However, the regulation of *Arc* during sleep has been previously shown to be a multi-level process with a balance between transcriptional changes, and changes in mRNA stability and proteasome degradation [79]. Arc protein has a short half-life (~ 37 to 47 min) with Arc protein stability regulated through ubiquitination and proteasome degradation [79–81]). Our research and that of others suggests that sleep deprivation increases transcription and translation of genes involved in protein degradation in the hippocampus (Additional files 1, 2 and [10]). Thus, potentially sleep deprivation impacts Arc protein stability in the hippocampus through increases in protein ubiquitination and degradation.

*Hspa5*, also known as *BiP*, is a conserved molecular chaperone that functions in multiple protein folding processes [82–84]. Previous research found no changes in the level of protein abundance after acute sleep deprivation [22], although *Hspa5* gene expression increases. Similar to the changes observed in the transcriptome, we found that *Hspa5* increased after sleep deprivation in the translatome. What explains these differences between mRNA levels and the lack of change in protein abundance? Hspa5 is a comparatively long-lived protein in the mouse brain with a half-life over 6 days [85]. Consequently, any changes in translation after 5 h of sleep deprivation are unlikely to be detected in studies of whole cell protein abundance.

### Differences in gene regulation between the transcriptome and translatome after sleep deprivation

We identified potentially significant functional differences between genes regulated at the level of the transcriptome and those regulated exclusively at the level of the translatome. Importantly, we identified genes for which translation was upregulated as well as genes that were downregulated strongly suggesting that sleep deprivation targets multiple mechanisms affecting translation. As previously discussed, sleep deprivation appears to decrease protein synthesis through decreased mTOR signaling and decreased phosphorylation of 4EBP2 [22]. However, mechanisms must also exist through which sleep deprivation can increase the translation of some transcripts independently of transcription. After transcription, mRNA molecules undergo multiple processing mechanisms that can affect the availability of these transcripts for translation, thus changing the rate of protein synthesis. Sleep deprivation could impact translation for specific RNA molecules through changes in mRNA processing via alternative splicing or polyadenylation, or sleep deprivation could alter mRNA degradation through changes in the nonsense-mediated decay pathway or miRNA mechanisms. mRNA interactions with RNA binding proteins can also affect the translation of mRNA. In the hippocampus, mRNA interactions with RNA binding proteins are important for the localization of mRNA transcripts necessary for synaptic plasticity [86]. Although identification of the mechanism(s) impacted by acute sleep deprivation that affect protein synthesis will undoubtedly be a subject of great interest in future research, we hypothesize that sleep deprivation targets multiple processes as the mechanisms that result in down-regulation of transcripts available for translation will be different than the mechanisms that can increase the availability of transcripts for protein synthesis.

Analysis of genes that were differentially regulated only through transcription revealed an enrichment of genes involved in transcriptional regulation. For example, the zinc finger transcription factor *Zmym1* mRNA is greatly increased in the transcriptome, but not at the level of the ribosome. Zmym1 has been recently shown to form part of a co-repressor complex that targets E-cadherin [87]. Potentially, these mRNA transcripts are sequestered or degraded in RNA–protein granules rendering them unavailable for translation. Cytoplasmic RNA granules including stress granules, rapidly form and dissociate in response to changing cellular conditions and can lead to translational repression [88–90]. In contrast, we found that the translational repressor *Sin3b* and the translation repressor *Eif4e2* were downregulated in the transcriptome but not changed by sleep deprivation in the TRAP-Seq data. These results suggest that these repressors are still functioning at similar levels after sleep deprivation contributing to eventual decreases in protein synthesis. Potentially, the transcriptional downregulation of these genes occurs in anticipation of a resumption of normal levels of protein synthesis during a future bout of recovery sleep.

Perhaps most importantly, our study identified genes that are differentially regulated in the translatome of excitatory neurons that had not been identified in studies of the transcriptome. Changes in gene regulation at the level of protein synthesis have the potential to rapidly affect cellular processes. Although our results suggest that these transcripts are regulated post-transcriptionally, we cannot rule out the possibility that the RiboTag selection for transcripts from excitatory neurons uncovered the differential expression of genes that may have changed in the transcriptome of excitatory neurons, but were masked by expression levels in other cell types such that the effect was diluted and not detected as significant. However, excitatory neurons comprise approximately 50% of the cells in the hippocampus and 85–90% of neurons [91] reducing the likelihood of this possibility. To minimize the chances of masking in our comparisons of gene regulation differences between the transcriptome and translatome, we focused on genes that were differentially regulated in the TRAP-Seq data, but not in the transcriptome, with an absolute difference in the magnitude of the fold change between the two data sets of at least 0.4.

As our experiments were centered on acute sleep deprivation, potentially some of the changes in gene regulation that were observed in the TRAP-Seq data are associated with an increase in sleep pressure at the cellular level. We found genes regulated in the TRAP-Seq data set were involved in cellular signaling, neuropeptide receptors, unfolded protein binding and circadian rhythms. Interestingly, we found that the core clock gene *Per1* was downregulated in the TRAP-Seq data, primarily due to the significant downregulation of the Per1-202 transcript. In both humans and animal models, circadian rhythms in the hippocampus appear necessary for memory formation and recall [92–97]. PER1 appears to play an important role in gating hippocampal memory formation [98, 99]. We also found that the core clock gene *Nr1d1* (commonly known as *Rev-Erbα*) was significantly downregulated in the TRAP-Seq data. Furthermore, we found that the arginine vasopressin receptor *Avpr1a,* one of three receptors for the circadian neuropeptide vasopressin*,* was downregulated. Avpr1a functions in the circadian synchronization of neurons in the SCN, the regulation of extra-SCN brain targets and appears involved in the timing of REM sleep [100–103], raising the possibility that one mechanism through which sleep deprivation disrupts hippocampal memory is through desynchronization of hippocampal neurons. Thus, the changes in gene regulation for circadian genes that we observed in the translatome have the potential to affect hippocampal memory through multiple mechanisms. Furthermore, hippocampal CA1 neurons exhibit circadian rhythms in excitability and in redox state adding additional links through which changes in circadian genes could affect neuronal function [104].

Although it can be difficult to distinguish the effects of circadian regulation from the effects of sleep deprivation on gene expression, recent research using genomic and proteomic approaches to identify regulation in synaptic compartments of the forebrain determined that the circadian clock regulated mRNA synaptic transcript levels while sleep, and sleep deprivation, affected the proteome [23, 24]. Acute sleep deprivation, and high sleep pressure, eliminated the daily oscillations in the synaptic proteome, while circadian oscillations in mRNA transcript abundance could still be detected, suggesting that sleep deprivation affects protein abundance through translational repression [24]. As in these studies of synaptic neurosomes, our TRAP-Seq results from hippocampal excitatory neurons also strongly suggest that sleep deprivation affects mRNA translation.

With more research investigating the effects of sleep deprivation, it has become clear that the effects of acute sleep deprivation vary between brain regions such as the hippocampus and cortex, and even between cell types or sub-regions [60, 105]. The effects of sleep deprivation on translation may also vary anatomically as sleep deprivation can cause region specific differences in the phosphorylation of translation factors involved in initiation [106] (reviewed in [107]). Our research demonstrates that in addition to anatomically based differences, acute sleep deprivation distinctly targets multiple levels of gene regulation. Moreover, acute sleep deprivation does not globally affect translation but rather governs the translation of subsets of genes with specific biological functions. Given the ever-increasing number of individuals affected by sleep deprivation and the association of sleep deprivation with cognitive impairments and neurodegenerative diseases, the need for increased research on the cellular impacts of sleep deprivation remains high. While the current study furthers our understanding of the impacts of sleep deprivation on gene regulation, future studies should consider the effects of acute and chronic sleep deprivation.

## Supplementary information

**Additional file 1: Figure S1.** Genes regulated by sleep deprivation in the transcriptome. *Arc* and *Hspb1* showed significantly increased expression in hippocampus following acute sleep deprivation in the transcriptome from the hippocampus, while *Arrdc4* was unchanged following sleep deprivation in the transcriptome. Unpaired t-test, *Arc*: p = 0.0010, *Hspb1*: p = 0.0023 and *Arrdc4*: p = 0.7291.

**Additional file 2: Table S1.** List of differentially expressed transcripts in the translatome after sleep deprivation. **Table S2.** List of differentially expressed isoforms in the translatome after sleep deprivation. **Table S3.** List of genes regulated only at the level of transcription between the microarray and TRAP data set. **Table S4.** List of genes regulated only at the level of translation between the microarray and TRAP data set. **Table S5.** List of qPCR primers used to study gene expression.

## Data Availability

The datasets generated and/or analysed during the current study are available in the NCBI's Gene Expression Omnibus repository, GEO Series accession GSE156925, https://www.ncbi.nlm.nih.gov/geo/query/acc.cgi?acc=GSE156925, and GEO accession GSE33302, https://www.ncbi.nlm.nih.gov/geo/query/acc.cgi?acc=GSE33302.
