# Supplementary material for: Translational changes induced by acute sleep deprivation uncovered by TRAP-Seq
Source: Mol Brain. 2020 Dec 3;13:165. doi: 10.1186/s13041-020-00702-5 (PMC7713217; doi:10.1186/s13041-020-00702-5)
Supplement: Supplementary file 1 — Additional file 1: Figure S1. Genes regulated by sleep deprivation in the transcriptome. Arc and Hspb1 showed significantly increased expression in hippocampus following acute sleep deprivation in the transcriptome from the hippocampus, while Arrdc4 was unchanged following sleep deprivation in the transcriptome. Unpaired t-test, Arc: p = 0.0010, Hspb1: p = 0.0023 and Arrdc4: p = 0.7291. [file 13041_2020_702_MOESM1_ESM.docx]

**Translational Changes Induced by Acute Sleep Deprivation**

**Uncovered by TRAP-Seq**

Lisa C. Lyons^1,2^, Snehajyoti Chatterjee^1^, Yann Vanrobaeys^1^, Marie E. Gaine^1^ and Ted Abel^1^

^1^Department of Neuroscience and Pharmacology, Iowa Neuroscience Institute, Carver College of Medicine, University of Iowa, Iowa City, Iowa

^2^Program in Neuroscience, Department of Biological Science, Florida State University, Tallahassee, Florida

**Figure S1**

**
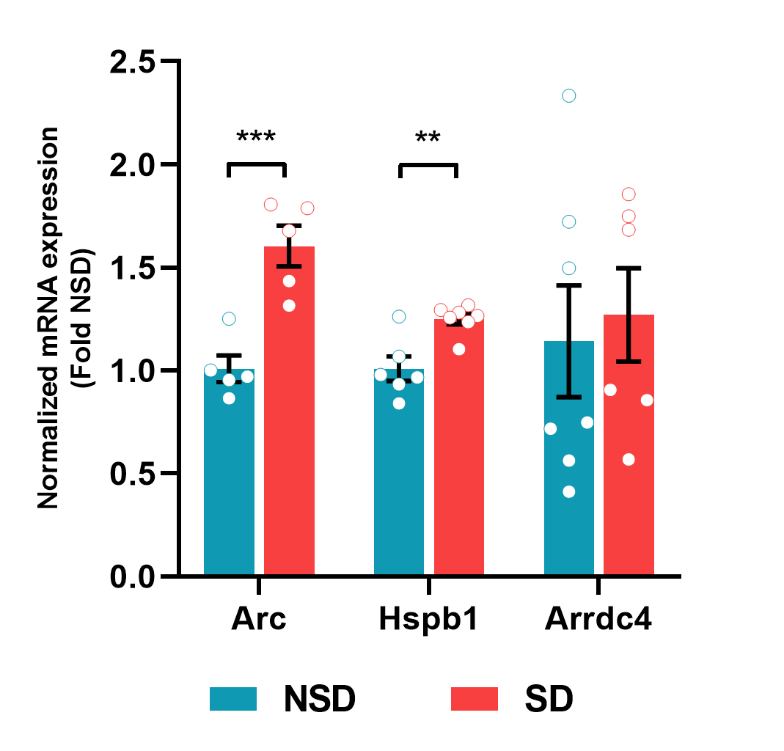
**
